# Editorial: Current trends and future prospects in the use of immunotherapy in ovarian cancer

**DOI:** 10.3389/fonc.2025.1737055

**Published:** 2025-11-18

**Authors:** Nikos G. Gavalas

**Affiliations:** 1 2nd Propaedeutic Department of Medicine, Medical School, National and Kapodistrian University of Athens- ATTIKON University Hospital, Athens, Greece; 2 Department of Clinical Therapeutics, Medical School, National and Kapodistrian University of Athens- Alexandra Hospital, Athens, Greece

**Keywords:** ovarian, cancer, immunotherapy, biomarkers, single cell

## Landscape and demand for immunotherapy in ovarian cancer

Ovarian cancer is the most prevalent cause of gynecological cancer in terms of mortality due to a number of factors, i.e., late-stage diagnosis, frequent relapse, and resistance to current treatment regimens, such as chemotherapy. Standard treatments, including surgical cytoreduction and platinum-based chemotherapy, tend to yield high initial response rates; however, the majority of advanced-stage patients experience disease recurrence, highlighting the urgent need for alternative therapies. In addition to other therapeutic modalities, immunotherapy has emerged as an innovative approach with the promising potential to improve outcomes by leveraging the immune system’s natural ability to recognize and attack tumor cells.

In recent years, the number of publications on ovarian cancer immunotherapy has been steadily increasing. At the same time, bibliometric analyses confirm the rapid expansion of this research field since 2019, as the discipline transitions from foundational trials to precision medicine and combination strategies.

## Tumor microenvironment and immune suppression

Key obstacles to the efficacy of immunotherapy in ovarian cancer include tumor heterogeneity combined with an immunosuppressive microenvironment. Ovarian tumors, in the majority of cases, are infiltrated by immune cells, including CD3+ and CD8+ T cells and macrophages. These populations are often functionally suppressed or reprogrammed by the tumor. Also, the upregulation of immune checkpoint molecules such as PD-1 and PD-L1, along with TGF-β and CTLA-4, impedes cytotoxic lymphocyte activity, enabling tumor evasion from immune surveillance (1).

Several studies have employed cutting-edge methods, such as single-cell sequencing, to profile the ovarian tumor immune microenvironment in detail (2). This approach sheds light on tumor cell subpopulations, immune cell phenotypes, and gene expression signatures that dictate immunologic dysfunction and therapy resistance. In this Research Topic, Zhao et al. report that succinic acid can reshape immune cell infiltration and function by targeting the hub genes SPP1, SLPI, and CD9, which could potentially disrupt tumor defenses.

## Advances in immunotherapeutic modalities

Immunotherapeutic strategies for ovarian cancer currently encompass targets such as checkpoint inhibitors (e.g., PD-1/PD-L1 antagonists), cell-based approaches (e.g., CAR-T and adoptive T cell transfer), and monoclonal antibodies. While immune checkpoint inhibitors have revolutionized the treatment of other malignancies, their clinical value in ovarian cancer is tempered by the low tumor mutational burden and limited immunogenicity; hence ovarian cancer is termed a “cold tumor” (3). Objective response rates remain modest, though subsets of patients with high PD-L1 expression may benefit more consistently (4). However, PD-L1 status is not a fully reliable predictor, as both PD-L1-positive and PD-L1-negative patients may respond to therapy, underscoring the complexity of the immune landscape.

Recent literature has explored alternative checkpoints (such as TIGIT, CD155, and DNAM-1) in combination therapies. These aim to enhance cytotoxic T cell and natural killer cell activity, increase tumor antigen presentation, and overcome suppression within the tumor microenvironment (1). Wang et al., in this Research Topic, along with other published literature, refer to multimodal approaches that include checkpoint inhibitors, targeted agents (e.g., PARP inhibitors), and antiangiogenic drugs (e.g., bevacizumab): all are under investigation to extend immunotherapeutic benefits.

## Platinum resistance mechanisms

Chemotherapy resistance stemming from platinum compound treatments remains a profound clinical challenge (5). Resistance mechanisms include altered cell death pathways, such as autophagy and immunosuppression orchestrated by COX-2 and tumor-associated macrophages (TAMs). Promising results have been generated by targeting the above; for example, TAM reactivity modulation may restore antitumor immunity and improve treatment outcomes, with a possible significant impact on patients with platinum-resistant disease.

## Biomarker discovery, patient stratification, and single-cell profiling

The discovery of robust predictive biomarkers is pivotal to optimizing immunotherapy. Several studies have focused on signatures of immune infiltration, tumor mutation burden, and molecular markers as tools for stratifying patients and customizing therapies. In previous articles (6) but also in this Research Topic, Gronauer et al. perform single-cell RNA sequencing, allowing for deep profiling of tumor heterogeneity, revealing population-specific tumor vulnerabilities and new therapeutic targets, and highlighting the necessity for precision medicine applications in this disease.

## Clinical trials and outcome data

Recent clinical trials validated targeted immunotherapy approaches, but they also underscored variable efficacy. While the administration of pembrolizumab, which is an anti-PD-1 agent, has demonstrated activity in some patients with high PD-L1 expression, overall survival improvements remain limited for the affected population as a whole. The presence of adverse events, including immune-related toxicities and autoimmune responses, should impose diligent risk management to preserve the quality of life of patients.

Targeted sequencing (7) and drug treatment combinations (1)— such as immune checkpoint inhibitors plus chemotherapy, targeted drugs, or alternative checkpoint blockades—represent major foci of contemporary research, and they aim to refine therapeutic windows and provide new ones, maximize efficacy, and reduce toxicity.

## Research trends, hotspots, and collaborative dynamics

Research trends in ovarian cancer show that research priorities have shifted from initial trials and monoclonal antibody development to topics such as CAR-T cell therapy and tumor microenvironment modulation. Influential research propels the field, guided by emerging insights from genomic databases and landmark trials focused on neoadjuvant and combination strategies (8).

Challenges for the future include treatment-induced toxicity and the slow clinical translation of genetic discoveries in order to improve survival rates.

## Future directions and opportunities

This editorial aims to highlight the seven contributions to this Research Topic that attempt to contribute to a rapidly maturing field committed to overcoming the limitations of immunotherapy in ovarian cancer. Further pathways to advance research include:

- Multidimensional tumor profiling via single-cell technologies and advanced bioinformatics, thus enabling tailored immunotherapeutic interventions.
- Continued exploration of combination therapies targeting immune checkpoints, with an emphasis on restoring immune cell function in resistant, immunosuppressing tumors.
- Enhanced biomarker identification and characterization, either as single molecules or combinations (signatures) to facilitate stratified clinical trial designs and personalized treatment algorithms.

Further coordinated efforts integrating basic science, translational research, and clinical expertise will accelerate innovation and broaden therapeutic accessibility.
